# Effect of Cost-Exemption Policy on Treatment Interruption in Patients With Newly Diagnosed Pulmonary Tuberculosis in South Korea

**DOI:** 10.34172/ijhpm.8262

**Published:** 2024-07-14

**Authors:** Sang Chul Lee, Jae Kwang Lee, Hyun Woo Ji, Jung Mo Lee, Seon Cheol Park, Chang Hoon Han

**Affiliations:** ^1^Division of Pulmonology and Allergy, Department of Internal Medicine, National Health Insurance Service Ilsan Hospital, Goyang, Republic of Korea.; ^2^Department of Research and Analysis, National Health Insurance Service Ilsan Hospital, Goyang, Republic of Korea.

**Keywords:** Tuberculosis, Compliance, Mortality, Risk factors, Administrative Data, Korea

## Abstract

**Background:**

In 2021, South Korea had the highest incidence rate (49 per 100 000 population) and the third highest mortality rate (3.8 per 100 000 population) due to pulmonary tuberculosis (TB) among Organization for Economic Co-operation and Development countries. Notably, premature interruption of TB treatment interferes with TB control efforts. Therefore, we examined the effect of the co-payment waiver on treatment interruption and mortality among patients with pulmonary TB in South Korea.

**Methods:**

Patients who had newly treated TB in South Korea from 2013 to 2019 were selected from the nationwide data of the entire Korean National Health Insurance Service (NHIS) population. The effects of policy implementation on treatment adherence and mortality rates depending on treatment interruption history were evaluated.

**Results:**

In total, 73 116 and 1673 patients with drug-susceptible (DS) and multidrug-resistant (MDR) pulmonary TB, respectively, were included in the final study population. After implementing the cost-exemption policy, the treatment interruption rate tended to decrease in the continuation phase in the DS-TB group (slope change: −0.097, *P*=.011). However, it increased in the intensive phase in the MDR-TB group (slope change: 0.733, *P*=.001). MDR-TB patients were likely to experience an interruption of TB treatment (adjusted odds ratio [aOR], 6.04; 95% CI, 5.43–6.71), and treatment interruption history was a significant risk factor for 1-year and overall mortality rates (adjusted hazard ratios [aHRs]: 2.01, 95% CI, 1.86–2.18 and 1.77, 95% CI, 1.70–1.84, respectively) in the DS-TB group.

**Conclusion:**

Implementing the cost-exemption policy effectively reduced the treatment interruption rate among patients with DS pulmonary TB.

## Background

**Key Messages**

**Implications for policy makers**

- Implementing the cost-exemption policy reduced the treatment interruption rate during the continuous phase of patients with drug-susceptible tuberculosis (DS-TB).
- Multidrug-resistant tuberculosis (MDR-TB) was identified as a risk factor for increasing treatment interruption, along with old age and multiple comorbidities.
- Efforts to improve patient adherence are necessary to lead to effective treatment outcomes in patients with TB.

**Implications for the public**

This study assessed the effect of introducing a cost-exemption policy on treatment interruption rates among pulmonary tuberculosis (TB) patients. Our results showed that the implemented policy reduced the treatment interruption rate in the continuation phase of the drug-susceptible TB (DS-TB) group. However, the benefit from the cost-exemption policy was not significant in the multidrug-resistant TB (MDR-TB) group. It is presumed to be due to the complex characteristics of MDR-TB treatment, such as the long treatment period and various drug side effects. Because treatment interruption history was a significant risk factor for 1-year and overall mortality rates in the DS-TB group, further efforts to improve the adherence of pulmonary TB patients are needed.

Although the incidence rate of tuberculosis (TB) decreased from 2015 to 2019, it remains one of the top 10 causes of death worldwide.^1,2^ The Korea Disease Control and Prevention Agency established the TB monitoring system and has been conducting the Public-Private Mix TB control project to reduce incidence rates to levels of developed countries through systematic TB prevention and control. However, TB remains a serious public health problem in South Korea,^3,4^ as South Korea had the highest incidence rate (49 per 100 000 population) and the third-highest mortality rate (3.8 per 100 000 population) due to TB among the Organization for Economic Co-operation and Development countries in 2021.^5,6^

 Premature interruption of TB treatment interferes with TB control efforts. This implies that the duration of exposure to TB drugs is insufficient for an effective cure. “A TB patient who did not commence treatment or whose treatment was interrupted for two consecutive months or more” is defined as a loss to follow-up (LTFU).^7-10^ The proportion of LTFU varies considerably among different countries, types of TB, and other patient populations; this has been studied extensively and was found to range from 2.5% to 44.9%.^11-16^ In South Korea, the LTFU rate has been reported to be as high as 5.4%–33.1%.^17-21^ As LTFU can lead to TB outbreaks and drug resistance, its reduction is essential to improve TB control.^22^ Various causes, such as low socioeconomic status, adverse effects of anti-TB medications, alcohol abuse, and marginalization, are related to the interruption of anti-TB medicines.^23-25^

 To improve the accessibility and provide the motivation for treating 138 rare and intractable disorders, including TB, the National Health Insurance Service (NHIS) of Korea initiated a co-payment reduction of up to 90% in 2007. Furthermore, to remove the individual economic burden and improve treatment compliance, co-payment for TB treatment was waived after July 2016.^5^

 However, no studies have evaluated the impact of the political change regarding the total exemption from medical service co-payment among patients with TB. Hence, this study aimed to investigate the difference in the treatment interruption rate of TB treatment and survival outcomes before and after the policy implementation.

## Methods

###  Data Source and Study Design

 All Korean residents must enroll in the Korean NHIS and receive a unique identification number at birth. Claims are accompanied by data regarding fully adjudicated medical and pharmacy claims in South Korea, including general demographic data, the 10th revision of the International Statistical Classification of Diseases (ICD-10) and Related Health Problems codes, medical institution type, medications prescribed, medical cost, and mortality information. This retrospective cohort study evaluated a nationwide data set comprising the entire Korean NHIS population from 2003 to 2019. We identified all outpatient visitors or hospitalized individuals with pulmonary TB during 2013–2019.

###  Case Identification

**Figure 1:**
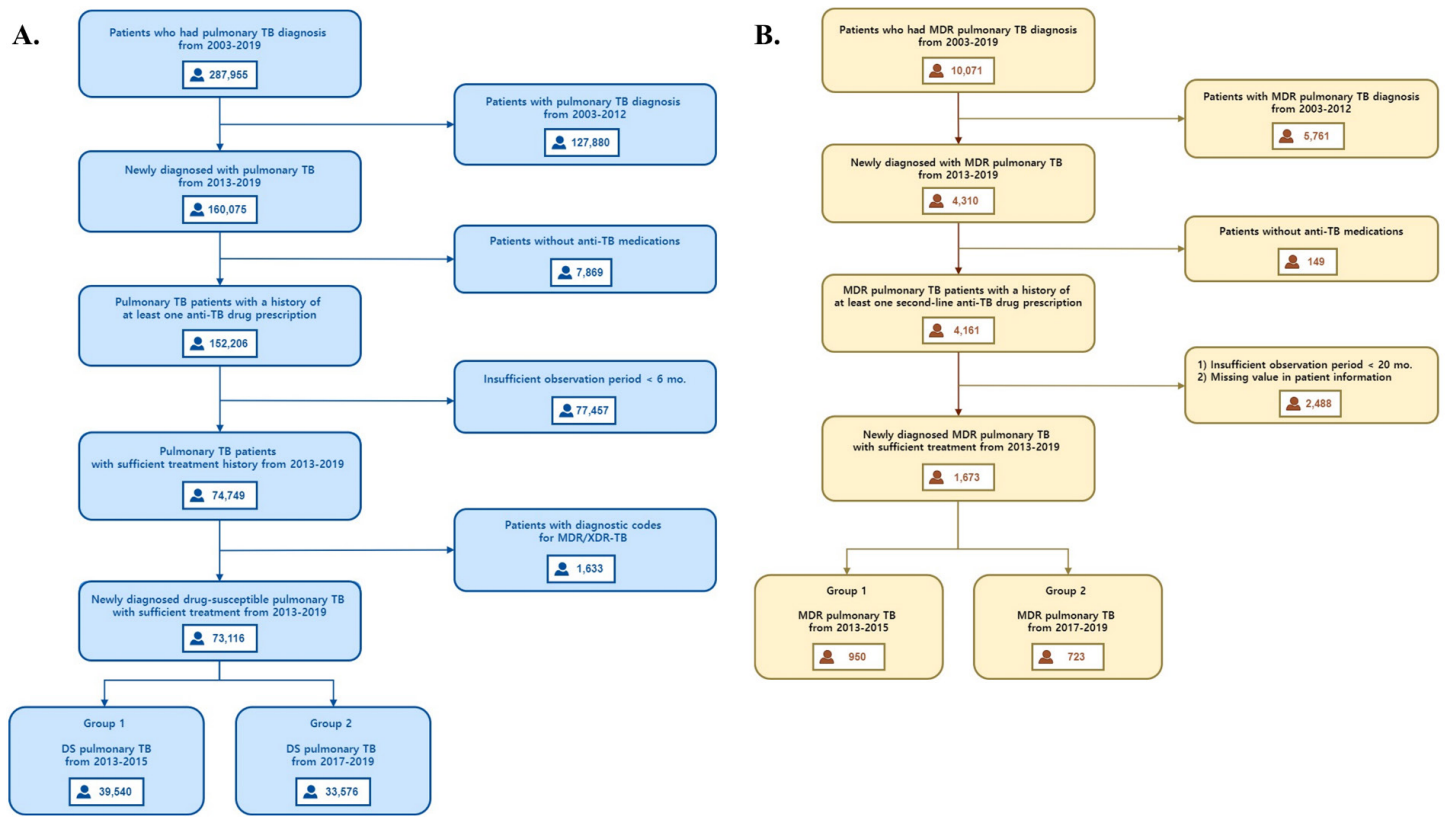
Flowchart of the Study Population. (A) Drug-susceptible pulmonary tuberculosis, (B) Multidrug-resistant pulmonary tuberculosis. Abbreviations: TB, tuberculosis; DS, drug-susceptible; MDR, multidrug-resistant; XDR, extensively drug-resistant.

 The schematic process for screening the study population is shown in Figure 1. Pulmonary TB cases were included only when diagnostic and medication codes were identified more than twice within the study period. Diagnostic codes for pulmonary TB were A15-16, U88.0-88.1, or U84.3, according to ICD-10. To better evaluate the effects of policy change, patients with newly diagnosed pulmonary TB were selected as study participants, and those with previous TB history or insufficient treatment duration (<6 months) were excluded. As the nature of the disease and treatment duration could differ from those of pulmonary and extra-pulmonary TB, patients with diagnostic codes for extra-pulmonary TB were also excluded. Anti-TB drug prescription(s) for initial screening included at least one of the following: isoniazid (INH), rifampicin (RIF), rifabutin, ethambutol, and pyrazinamide. Cases of patients who had both diagnostic codes for multidrug-resistant (MDR) or extensively drug-resistant (XDR) TB and prescription(s) for two or more second-line anti-TB medications were defined as MDR pulmonary TB cases. A list of anti-TB medications is shown in Table S1 (Supplementary file 1). The study period was divided into pre- and post-cost-exemption periods based on the policy implementation date (July 2016). Subjects were included in the study group if their TB diagnosis date, treatment start date, and end date were all within the same period. Although the date of TB diagnosis and the start of treatment were included in the pre-exemption period, subjects who completed treatment after 2016 were excluded from the study. Finally, study participants were grouped according to the presence of drug resistance for first-line TB drugs (drug-susceptible [DS] TB or MDR-TB) and treatment period (pre- or post-exemption).

###  Charlson Comorbidity Index

 The Charlson comorbidity index (CCI) is a widely used prognostic model that predicts 1-year mortality risk depending on individual comorbidities. Each comorbidity was scored, and the CCI was calculated by summing the comorbidity scores (Table S2). This variable was adapted because it is useful for measuring the effect of comorbidities on mortality using the administrative database, including ICD-10 codes.^26,27^

###  Definition of Treatment Phase and Treatment Interruption 

 Treatment periods for treating pulmonary TB are divided into the intensive and continuation phases. The treatment regimen for DS pulmonary TB consists of a 2-month intensive phase followed by a 4 or 7-month continuation phase. For treatment of DS pulmonary TB, a regimen consisting of 2 months of INH, RIF, pyrazinamide, and ethambutol followed by a continuation phase of 4 months of INH and RIF.^28^ In patients with MDR-TB, a total treatment duration of 18–20 months is recommended for most patients, with an intensive phase of 6–7 months and a continuation phase of 12 months or more. During the intensive phase, using at least five drugs consisting of an injectable agent (other than imipenem–cilastatin or meropenem) is recommended.^29^ Treatment interruption was defined as an interruption of anti-TB treatment of at least two consecutive weeks during the intensive phase or at least 2 consecutive months during the continuation phase.^30-32^

###  Outcome Measures

 The primary outcome was the difference in the treatment interruption rates among patients with pulmonary TB before and after policy implementation. The secondary outcomes included 1-year and overall mortality among the study groups and risk factors for predicting treatment interruption of anti-TB therapy and all-cause mortality.

###  Statistical analysis 

 The variables of each group were compared using the paired *t* test or chi-square test. An interrupted time series (ITS) analysis was used to evaluate the longitudinal impact of introducing the cost-exemption policy. ITS is regarded as one of the most robust quasi-experimental designs to assess the impact of an intervention and has been used in numerous studies.^33,34^ In ITS analysis, data are arranged at evenly spaced time intervals and separated by the intervention into segments. Then, the ITS analysis assesses the short-term impact of the intervention, as measured by a change in the level, and the over-time effect, measured by a change in the trend (ie, slope), after the intervention.^35^ The time point variable represents the entire observation period in 1-month units, and a dummy variable was created and used by setting the time point before policy intervention to 0 and the time point after policy intervention to gender, age, premium quintile, and CCI score were used as correction variables. Cox proportional hazard models were fit to estimate the all-cause adjusted hazard ratio (aHR) and 95% confidence interval (CI).^13^ Subsequently, multivariate logistic regression analysis was performed to evaluate the association between risk factors and treatment interruption of pulmonary TB. The results are reported as the adjusted odds ratio (aOR) with a 95% CI. All statistical analyses were performed using SAS version 9.4 (SAS Institute, Inc., Cary, NC) at a significance level of 5%.

## Results

###  Study Population

 Data from 73 116 and 1673 patients with DS pulmonary TB and MDR pulmonary TB, respectively, were extracted during the study period. Among the patients with DS pulmonary TB, 39 540 and 33 576 were newly diagnosed and treated before and after introducing the total cost exemption of medical service co-payment, respectively. In the case of MDR-TB, newly diagnosed patients before and after introducing the exemption policy were 950 and 723 for each, respectively (Figure 1). Male and older patients are more common in both groups. In addition, non-metropolitan residents and patients with multiple comorbidities accounted for a large portion of the study population (Table).

**Table:** Baseline Characteristics of the Study Population

**Characteristics**	**DS Pulmonary TB**			**MDR Pulmonary TB**		
	**Pre-cost Exemption** **(n = 39 540)**	**Post-cost Exemption** **(n = 33 576)**	***P *** **Value**	**Pre-cost Exemption** **(n = 950)**	**Post-cost Exemption** **(n = 723)**	***P *** **Value**
Age (y)	54.5 ± 19.8	59.7 ± 19.2	<.0001	45.9 ± 16.2	50.1 ± 17.1	<.0001
<20	1266 (3.2)	591 (1.7)		21 (2.2)	12 (1.6)	
20–39	8819 (22.3)	5346 (15.9)		333 (35.0)	202 (27.9)	
40–59	12 647 (31.9)	9609 (28.6)		406 (42.7)	308 (42.6)	
≥60	11 388 (42.5)	18 030 (53.7)		190 (20.0)	201 (27.8)	
Gender						
Male	23 071 (58.3)	20 034 (59.6)	<.0001	632 (66.5)	495 (68.4)	.402
Female	16 469 (41.6)	13 542 (40.3)		318 (33.4)	228 (31.5)	
Residential area						
Metropolitan	17 105 (43.2)	14 035 (41.8)	<.0001	449 (47.2)	320 (44.2)	.222
Non-metropolitan	22 435 (56.7)	19 541 (58.2)		501 (52.7)	403 (55.7)	
CCI						
0	5987 (15.1)	3914 (11.6)	<.0001	145 (15.2)	115 (15.9)	.009
1	9828 (24.8)	6991 (20.8)		237 (24.9)	160 (22.1)	
2	8178 (20.6)	6671 (19.8)		242 (25.4)	148 (20.4)	
≥3	15 547 (39.3)	16 000 (47.6)		326 (34.3)	300 (41.4)	
Treatment interruption	11 905 (30.1)	8515 (25.3)	<.0001	649 (68.3)	505 (69.8)	.502
Multidrug-resistance						
MDR-TB	N/A	N/A	N/A	853 (89.7)	648 (89.6)	.913
XDR-TB	N/A	N/A		97 (10.2)	75 (10.3)	

Abbreviations: CCI, Charlson comorbidity score; DS, drug-susceptible; MDR, multidrug-resistant; TB, tuberculosis; XDR, extensively drug-resistant; N/A, not available. Notes: Data are presented as means ± standard deviations and numbers (%).

###  Treatment Interruption by Treatment Phase 

**Figure 2:**
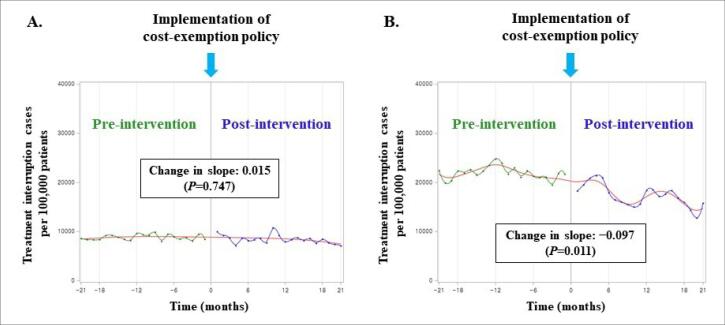
The Treatment Interruption Rate Before (Green Line) and After (Blue Line) the Cost-Exemption Policy Among Patients With Drug-Susceptible Tuberculosis. The ITS model was fit through regression analysis, and gender, age, premium quintile, and CCI score were used as correction variables. (A) Intensive phase (initial 2 months period), (B) Continuation phase (period after 2 months). Abbreviations: ITS, interrupted time series; CCI, Charlson comorbidity index.

**Figure 3:**
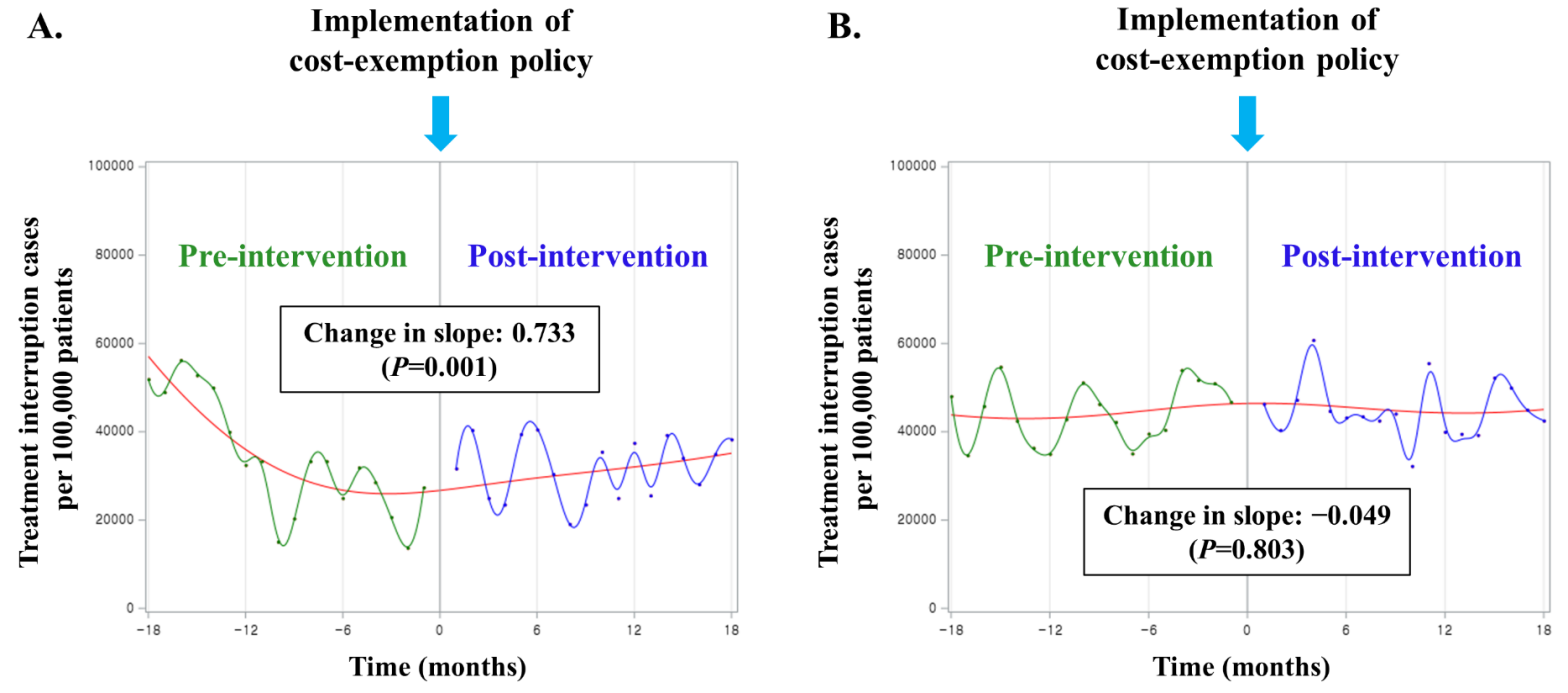
The Treatment Interruption Rate Before (Green Line) and After (Blue Line) the Cost-Exemption Policy Among Patients With Multidrug-Resistant Tuberculosis. The ITS model was fit through regression analysis, and gender, age, premium quintile, and CCI score were used as correction variables. (A) Intensive phase (initial 6 months period), (B) Continuation phase (period after 6 months). Abbreviations: ITS, interrupted time series; CCI, Charlson comorbidity index.

 In cases of DS pulmonary TB, the treatment interruption rate during the post-policy period was lower than before the policy change (30.1% vs. 25.3%, *P*< .0001). However, the treatment interruption rate of anti-TB medications in MDR pulmonary TB showed no difference between before and after introducing the exemption policy (68.3% vs. 69.8%, *P =*.502) (Table). Compared to before and after introducing the exemption policy, the number of treatment interruption cases per 100 000 patients during the intensive phase showed no significant difference among the patients with DS-TB (8841 vs. 8422, *P =*.074). Similarly, there was no significant change in the slope of the treatment interruption rate (slope change: 0.015, *P =*.747). In contrast, in the continuation phase, the number of treatment interruption cases per 100 000 patients and trend slope significantly decreased after the cost-exemption policy (21 940 vs. 17 319, *P*< .0001; slope change: −0.097, *P =*.011) (Figure 2). Although the number of treatment interruption cases per 100 000 patients during the intensive phase of patients with MDR-TB decreased (34 182 vs. 31 784, *P =*.501), the slope showed an increasing tendency after the policy change (slope change: 0.733, *P =*.001). Contrarily, no significant change occurred during the continuation phase (44 348 vs. 44 962, *P =*.782; slope change: −0.049, *P =*.803) (Figure 3).

###  Risk Factors for TB Treatment Interruption

 Old age (aOR, 1.15; 95% CI, 1.11–1.19), multiple comorbidities (CCI ≥3; aOR, 1.17; 95% CI, 1.13–1.21), and drug resistance to first-line TB drugs (aOR, 6.04; 95% CI, 5.43–6.71) were revealed as risk factors for predicting the treatment interruption in the study population (Table S3). They were also identified as significant risk factors in the analysis among patients with DS-TB and those with MDR-TB (Tables S4 and S5).

###  Mortality Rates According to the TB Treatment Interruption

**Figure 4:**
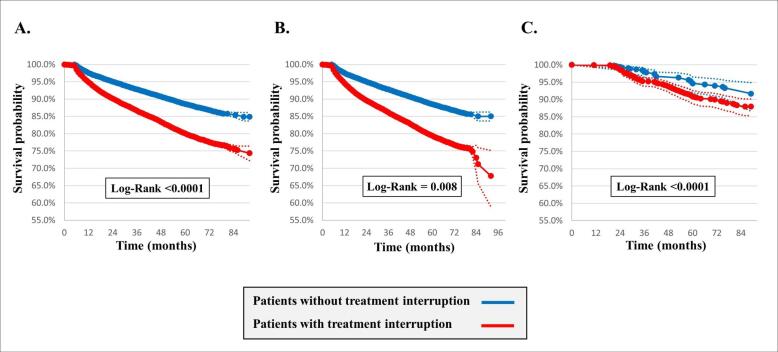
Kaplan–Meier Survival Curve for All-Cause Mortality Among Patients With Pulmonary Tuberculosis. (A) Total study population, (B) Drug-susceptible tuberculosis, (C) Multidrug-resistant tuberculosis.

 The mean follow-up periods were 47.9 and 63.8 months for DS-TB and MDR-TB, respectively. In the case of DS pulmonary TB, patients with treatment interruption showed a higher overall mortality rate than those without treatment interruption (32.2% vs. 15.0%, *P <*.0001) (Figure 4). As for MDR-TB, mortality was also higher in patients with a history of treatment interruption (12.1% vs. 8.3%, *P =*.008).

###  Risk Factors Associated With 1-Year and Overall Mortality 

**Figure 5:**
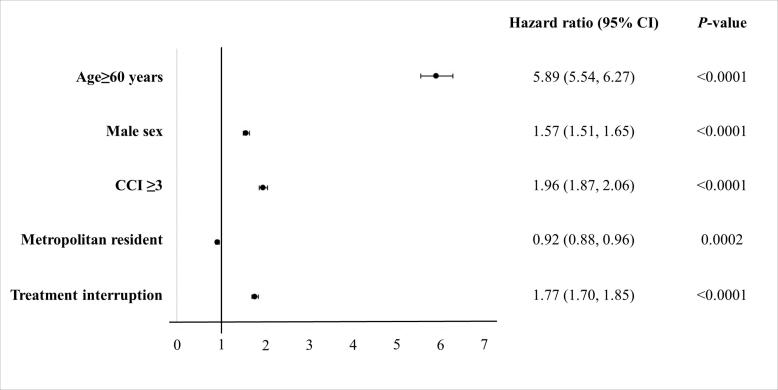
Risk Factors Associated With Overall All-Cause Mortality in Patients With ‎Drug-Susceptible Tuberculosis. Abbreviations: CCI, Charlson comorbidity index; CI, ‎confidence interval.‎

 Old age (aHR 5.97; 95% CI, 5.24–6.80 [1-year mortality], aHR, 5.89; 95% CI, 5.54–6.27 [overall mortality]); male sex (aHR 1.47; 95% CI, 1.35–1.60 [1-year mortality], aHR, 1.57; 95% CI, 1.51–1.65 [overall mortality]); high CCI (aHR 2.40; 95% CI, 2.18–2.65 [1-year mortality], aHR, 1.96; 95% CI, 1.87–2.06 [overall mortality]); and history of treatment interruption (aHR 2.01; 95% CI, 1.86–2.18 [1-year mortality], aHR, 1.77; 95% CI, 1.70–1.85 [overall mortality]) were revealed as risk factors for both 1-year and overall mortality among the patients in the DS-TB group (Figure 5 and S1).

 In the MDR-TB group, old age (aHR 3.98; 95% CI, 2.74–5.79), male sex (aHR 2.90; 95% CI, 1.81–4.64), and high CCI (aHR 2.53; 95% CI, 1.71–3.74) were significant risk factors for increasing overall mortality (Figures S1 and S2).

## Discussion

 In this study, we examined the effect of the co-payment waiver on treatment interruption and mortality among patients with pulmonary TB in South Korea. Many countries have implemented or tried to implement universal health coverage to improve drug adherence and treatment outcomes of pulmonary TB.^36-40^ In this context, the government of South Korea waived the medical service co-payment for pulmonary TB following July 2016. When comparing characteristics of the study population from before and after the policy change, the patient’s age tended to increase after the cost exemption, and the proportion of non-metropolitan residents increased compared to metropolitan residents in both DS-TB and MDR-TB groups. It is possible that elderly patients with financial burdens actively underwent diagnostic testing and received treatment compliantly after the co-payment waiver. A decreasing trend of the treatment interruption rate during the continuation phase in DS-TB was also observed. This suggests that reducing the financial burden could help increase treatment compliance because of the nature of TB, which requires more than six months of treatment. Meanwhile, various TB control policies were also established and implemented to improve the quality of TB management during the study period. After introducing a public-private mix collaboration pilot project in 2007, it was expanded to 128 hospitals in 2016. Positive public-private mix outcomes have been reported in TB control in terms of increasing the treatment success rate in South Korea.^41,42^ The government has also increased budgets and strengthened patient management policies, including expanding comprehensive TB patient management, providing high-quality diagnosis and treatment, contact investigation, and latent TB infection treatment, to strengthen the TB control program since 2011.^43,44^ Thus, the reduction in treatment interruption rate is not a single effect of cost exemption but a combined effect of various TB control policies.

 Our findings revealed that treatment interruption during the intensive phase was not significantly different between the two periods. The most important step in the intensive phase of TB treatment is to reduce the spread of TB in the community by making the patient non-infectious.^45^ Furthermore, treatment compliance during the intensive phase is essential because early treatment interruption is known to be a risk factor for poor treatment outcomes.^46-48^ Causes of non-adherence during the intensive phase include not only a financial burden but also side effects of drugs, level of education, living environment, and absence of caregivers.^49,50^ Therefore, interventions to increase compliance with TB treatment should include directly observed therapy, digital health technologies (eg, video-observed treatment), and education strategies, as well as policies to reduce the cost burden.^48,51^

 Similarly, we did not find a significant difference in the treatment interruption rate after the cost exemption among patients with MDR-TB. The complex nature of MDR-TB treatment, such as the long duration of treatment and high rate of adverse events of second-line TB drugs, contributes to lower compliance than first-line TB treatment.^52^ In addition, various individual and psycho-social supports, such as self-motivation, awareness concerning disease and treatment, counseling support, family support, and nutritional support, were important drivers for the successful treatment of MDR-TB.^53-55^ Therefore, considering and correcting these factors is expected to reduce treatment interruption and improve treatment outcomes.

 Unmodifiable variables, such as old age, multiple comorbidities, and MDR-TB, were revealed as risk factors for treatment interruption. Notably, patients with MDR-TB were 6.04 times more likely to experience treatment interruption than those with DS-TB. Treatment interruption, including LTFU, leads to prolonged infectiousness, relapse, death, acquired drug resistance, and treatment failure; thus, special attention is needed during treatment for patients with risk factors.^56^

 According to a long-term mortality analysis study using nationwide population-based data in South Korea, the 5-year mortality rate for TB infection was 24.7%, and the overall mortality rate was 3.23 times higher than that of the general population.^57^ Host factors, such as age, sex, bacteriological status, comorbid conditions, immune and nutritional status, and substance abuse, have been identified as risk factors for death in patients with TB.^58^ As our findings suggest that the mortality rate in patients with DS-TB undergoing treatment interruption was 1.77 times higher than the corresponding in those without, efforts to increase treatment compliance of TB are significant and urgent to improve the survival rate.

 Our study had several strengths. To our knowledge, this is the first study to analyze the effect of implementing the cost-exemption policy on the clinical outcomes of pulmonary TB. In particular, a large-scale study using national health insurance data strengthens the power of the results. Through this study, we found that the political change to widen the coverage helped improve the treatment compliance of patients with pulmonary TB. It can also be applied to improve treatment outcomes of other refractory diseases. In addition, the experience of treatment interruption of anti-TB medications, regardless of treatment termination status, negatively affects the long-term survival rate; thus, efforts to increase treatment compliance should be highlighted.

 However, this study also has some limitations. As pulmonary TB cases were screened and analyzed based on the operational definition using diagnostic codes rather than nationwide TB notification data, we could not confirm the four treatment outcomes according to the guidelines and previous studies.^1,59^ Considerably, information on a sputum smear or culture status was unavailable in our data; hence, we could not capture detailed treatment outcomes, such as treatment failure. It is possible that patients who had completed anti-TB treatment for >6 months but had a recurrence of TB afterward or who were judged as treatment failure and restarted anti-TB drugs were also included as treatment disruption. In addition, it was challenging to distinguish between treatment completion and LTFU; therefore, the treatment interruption rate reported herein was likely higher than the LTFU rate in the actual central reported data. A recent study analyzing the ratio of LTFU in patients with DS-TB from the Korean National TB Surveillance System reported the LTFU rate as 4.4%–12.3%, supporting this theory.^60^ In addition, with the operational definition, it was difficult to distinguish patients with TB who had mono-resistance to the first-line regimen. Hence, some patients with mono-resistant TB could be included in both groups, which might have affected the results of treatment interruption and mortality.

## Conclusion

 Implementing the cost-exemption policy reduced the treatment interruption rate during the continuous phase of patients with DS-TB. Additionally, MDR-TB was identified as a risk factor for increasing treatment interruption, along with old age and multiple comorbidities (CCI ≥3). As the history of treatment interruption, regardless of the treatment termination status, was a risk factor for increased mortality, efforts to improve patient adherence would be necessary to lead to effective treatment outcomes in patients with TB.

## Acknowledgements

 This study used the National Health Information Database (NHIS-2022-1-460), made by NHIS. The authors alone are responsible for the content and writing of the paper.

## Ethical issues

 The Institutional Review Board of NHIS Ilsan Hospital approved the study, and the study adhered to the Declaration of Helsinki’s tenets (NHIMC 2022-05-015). As this study was based on anonymous health claims data, the requirement for patient consent was waived.

## Competing interests

 Authors declare that they have no competing interests.

## Supplementary files

Supplementary file 1 contains Tables S1-S5 and Figures S1-S2.
