# Supplementary material for: Effect of Cost-Exemption Policy on Treatment Interruption in Patients With Newly Diagnosed Pulmonary Tuberculosis in South Korea
Source: Int J Health Policy Manag. 2024 Jul 14;13:8262. doi: 10.34172/ijhpm.8262 (PMC11365073; doi:10.34172/ijhpm.8262)
Supplement: Supplementary file 1 — contains Tables S1-S5 and Figures S1-S2. [file ijhpm-13-8262-s001.pdf]

Article title: Effect of Cost-Exemption Policy on Treatment Interruption in Patients With Newly Diagnosed Pulmonary Tuberculosis in South Korea

Journal name: International Journal of Health Policy and Management (IJHPM)

Authors' information: Sang Chul Lee[1]*, Jae Kwang Lee[2], Hyun Woo Ji[1], Jung Mo Lee[1], Seon Cheol Park[1], Chang Hoon Han[1]

[1]Division of Pulmonology and Clinical Allergy, Department of Internal Medicine, National Health Insurance Service Ilsan Hospital, Goyang, Republic of Korea.

[2]Department of Research and Analysis, National Health Insurance Service Ilsan Hospital, Goyang, Republic of Korea.

***Correspondence to:** Sang Chul Lee; Email: sc218@nhimc.or.kr

Citation: Lee SC, Lee JK, Ji HW, Lee JM, Park SC, Han CH. Effect of cost-exemption policy on treatment interruption in patients with newly diagnosed pulmonary tuberculosis in South Korea. Int J Health Policy Manag. 2024;13:8262. doi:10.34172/ijhpm.8262

Supplementary file 1

Table S1. List of anti-tuberculosis drugs

Drug	Dosage formulation(s)
1. First-line drugs	
- Isoniazid (H)	Tablet
- Rifampicin, Rifabutin (R)	Tablet / Capsule
- Ethambutol (E)	Tablet
- Pyrazinamide (Z)	Tablet
- Fixed dose combination (HRZE / HRE)	Tablet
2. Second-line drugs	
- Fluoroquinolones<br>: Levofloxacin (Lfx), moxifloxacin (Mfx)	Tablet, IV injection
- Bedaquiline (Bdq)	Tablet
- Linezolid (Lzd)	IV injection
- Cycloserine (Cs)	Capsule
- Clofazimine (Cfz)	Capsule
- Aminoglycosides<br>: Streptomycin (S), kanamycin (Km), amikacin (Am)	IV/IM injection
- Carbapenems<br>: Meropenem (Mpm), imipenem (Ipm)	IV injection
- Para-aminosalicylate (PAS)	Granules
- Prothionamide (Pto)	Tablet
- Delamanid (Dlm)	Tablet

IV, intravenous; IM, intramuscular

Table S2. ICD-10 codes for the Charlson comorbidity index

Disease	Score	ICD-10 codes
Myocardial infarction	1	I21; I22; I23; I25.2; I25.3;
Congestive heart failure	1	I50; I11.0; I13.0; I13.2; I25.5;
Peripheral vascular disease	1	I70-I74; I731; I738; I739; I77; I771; I790; I792; K551; K558; K559; Z958; Z959; R02; K55.003; K55.004; K55.010;
Cerebrovascular disease	1	I60-I63; I65; I66; G450-G452; G458; G459; G46; I64; G454; I670; I671; I672; I674; I675; I676; I677; I678; I679; I681; I682; I688; I69; I60-I69; G45; G46;
Dementia	1	F00; F01; F02; F03; F051; G30; G311;
Chronic pulmonary disease	1	J40-J47; J67; J60-J66; I278; I279; J684; J701; J703; J84.1; J92.0; J96.1; J98.2; J98.3; J84.9;
Connective tissue disease	1	M30-M36; M332; M331; M053; M058-M060; M063; M069; M050-M052; M353; M05; M06; M315; M351; M360; M08; M09; D86;
Ulcer disease	1	K25-K28; K22.1;
Mild liver disease	1	B18; K700-K703; K709; K713; K715; K717; K73; K74; K760; K762-K764; K768; K769; Z944; K71.0; K71.6; K71.9; K75; K76.103; B16.9; B19.9;
Diabetes mellitus	1	E109-E111; E119; E139; E101; E131; E141; E135; E100; E11.600; E120; E121; E126; E128-E131; E136; E138-E141; E146; E148; E149; E11.701;
Hemiplegia	2	G81; G041; G820-G822; G114; G801; G802; G82; G830; G831-G834; G839; G838;
Moderate/severe renal	2	N03; N052; N053; N054; N055; N056; N072; N073; N074;

disease		N01; N18; N19; N25; I120; I131; N057; Z490; Z491; Z492; Z940; Z992/I12; I13; N00-N05; N07; N11; N14; N17; Q61;
Diabetes mellitus with chronic complications	2	E102; E112; E132; E142; E103; E113; E133; E143; E104; E114; E134; E144; E105; E107; E115; E122-E125; E127; E135; E137; E145; E147; E10.6; E10.8; E11.601; E11.8; E11.6; E14.5;
Any tumor	2	C00-C76;
Leukemia	2	C91-C95; C91.001; C95.902;
Lymphoma	2	C81-C85; C88; C90; C96;
Moderate/severe liver disease	3	K729; K76-K767; K721; I850; I859; I864; I982; K704; K711; B15.0; B15.9; B16.0; B16.2; B19.0; K72; I85; Z944;
Metastatic solid tumor	6	C77-C80;
AIDS	6	B20; B21; B22; B23; B24;

AIDS, acquired immune deficiency syndrome; ICD-10, 10th revision of the International Statistical Classification of Diseases and Related Health Problems

Table S3. Risk factors associated with treatment interruption in the total study population

Variables	Univariate analysis		Multivariate analysis	
	Crude OR (95% CI)	*P*-value	Adjusted OR (95% CI)	*P*-value
Age ≥60 years	1.17 (1.13–1.21)	<0.0001	1.15 (1.11–1.19)	<0.0001
Male sex	0.98 (0.95–1.01)	0.129	0.98 (0.95–1.01)	0.154
CCI ≥3	1.22 (1.18–1.26)	<0.0001	1.17 (1.13–1.21)	<0.0001
Metropolitan resident	0.96 (0.93–0.99)	0.005	0.97 (0.94–1.00)	0.051
Multidrug-resistance (MDR or XDR-TB)	5.74 (5.17–6.37)	<0.0001	6.04 (5.43–6.71)	<0.0001

CCI, Charlson comorbidity index; MDR, multidrug-resistant; OR, odds ratio; TB, tuberculosis; XDR, extensively drug-resistant

Table S4. Risk factors associated with treatment interruption of anti-TB treatment in patients with drug-susceptible TB

Variables	Univariate analysis		Multivariate analysis	
	**Crude OR (95% CI)**	***P*-value**	**Adjusted OR (95% CI)**	***P*-value**
Age ≥60 years	1.22 (1.19–1.26)	<0.0001	1.14 (1.10–1.18)	<0.0001
Male sex	0.96 (0.93–0.99)	0.013	0.98 (0.95–1.01)	0.151
CCI ≥3	1.24 (1.20–1.28)	<0.0001	1.17 (1.12–1.21)	<0.0001
Metropolitan resident	0.95 (0.92–0.98)	0.0008	0.96 (0.93–1.00)	0.030

CCI, Charlson comorbidity index; CI, confidence interval; OR, odds ratio; TB, tuberculosis

Table S5. Risk factors associated with treatment interruption of anti-TB treatment in patients with MDR-TB

Variables	Univariate analysis		Multivariate analysis	
	**Crude OR (95% CI)**	***P*-value**	**Adjusted OR (95% CI)**	***P*-value**
Age ≥60 years	1.51 (1.17–1.96)	0.001	1.40 (1.07–1.84)	0.016
Male sex	0.98 (0.79–1.23)	0.876	0.98 (0.78–1.22)	0.842
CCI ≥3	1.34 (1.08–1.67)	0.008	1.23 (0.98–1.55)	0.080
Metropolitan resident	1.10 (0.89–1.36)	0.364	1.14 (0.93–1.41)	0.216
XDR-TB	0.62 (0.45–0.86)	0.004	0.63 (0.45–0.87)	0.005

CCI, Charlson comorbidity index; CI, confidence interval; OR, odds ratio; TB, tuberculosis; XDR-TB, extensively drug-resistant tuberculosis

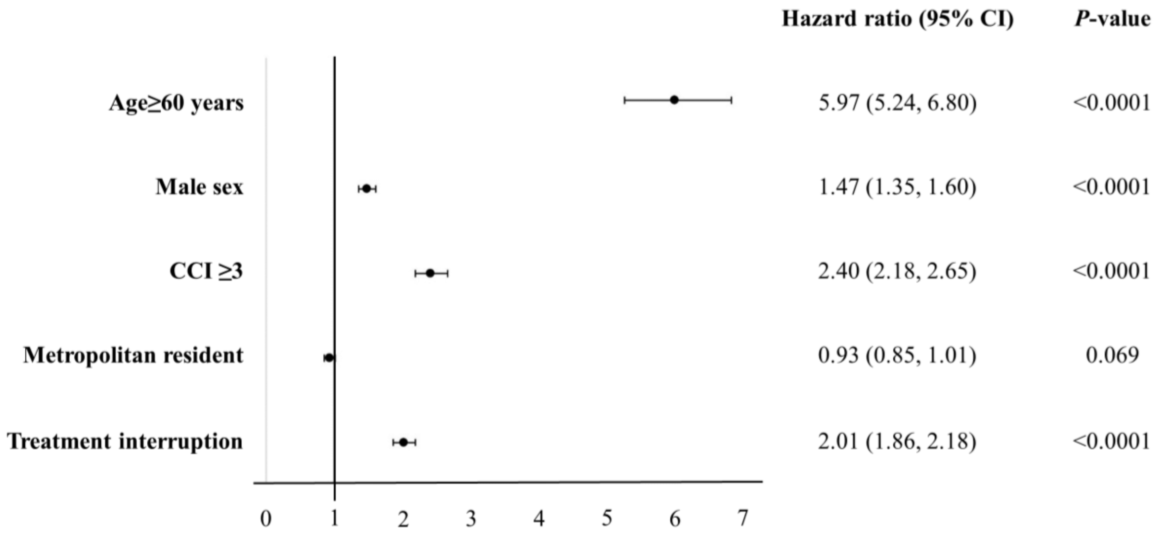

Figure S1. Risk factors associated with 1-year all-cause mortality in patients with drug-susceptible TB

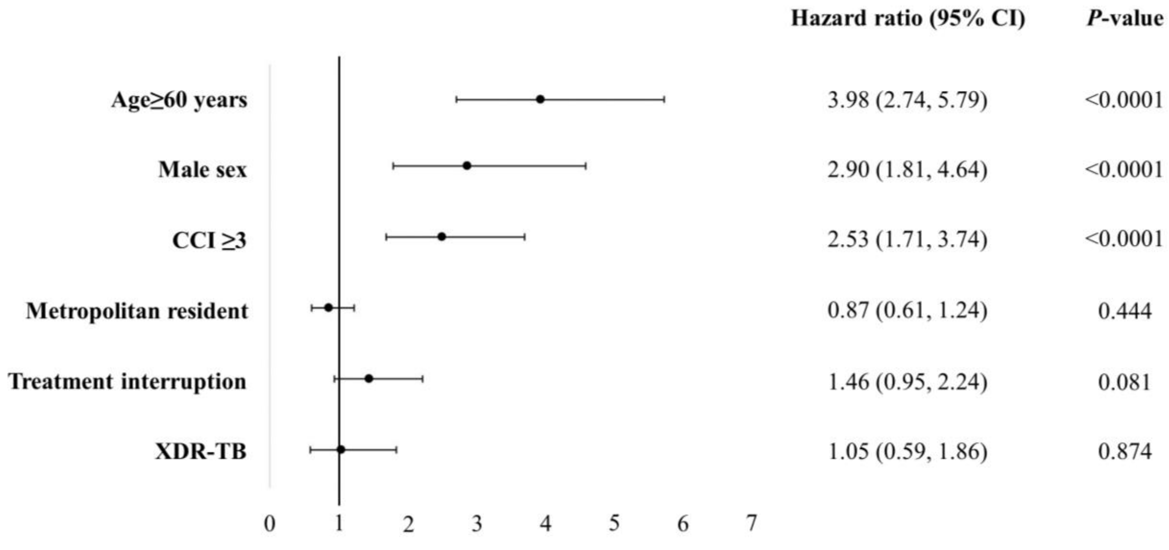

Figure S2. Risk factors associated with overall all-cause mortality in patients with MDR-TB
